# Association between spexin and type 2 diabetes mellitus: a systematic review and meta-analysis

**DOI:** 10.3389/fendo.2026.1792528

**Published:** 2026-04-10

**Authors:** Xin Sun, Jiayi Yao, Jinsong Kuang, Caihong Xin

**Affiliations:** 1 Department of Endocrinology, First Affiliated Hospital of Soochow University, Suzhou, China; 2 Department of Endocrinology, Fourth People`s Hospital of Shenyang, Shenyang, China

**Keywords:** meta, meta-analysis, spexin, T2DM, type 2 diabetes mellitus

## Abstract

**Background:**

Accumulating evidence suggests that spexin is implicated in cellular energy balance, glucose and lipid metabolism, appetite suppression, and water-electrolyte regulation, and is closely associated with metabolic conditions including obesity, hyperglycemia, and hyperlipidemia. However, due to limitations in sample size, ethnic diversity among study populations, and variability in clinical study designs, existing findings on the association between spexin and Type 2 diabetes mellitus (T2DM) remain inconsistent.

**Aim:**

This meta-analysis aimed to statistically evaluate the level of spexin in patients with T2DM.

**Methods:**

A systematic literature search was conducted across five electronic databases (PubMed, Web of Science, OVID, Elsevier Science Direct, and Wiley Online Library). The search strategy targeted the terms “spexin” in conjunction with “Type 2 diabetes mellitus” or “T2DM” in title and abstract fields. Results are presented as standardized mean differences (SMD) with 95% confidence intervals (CI).

**Results:**

Eleven articles (1,122 cases and 681 controls) were included in the meta-analysis. The results of the meta-analysis indicated that the circulating spexin in patients with T2DM was significantly lower than that of the controls (SMD: -2.32, 95% CI: -3.32, -1.31).

**Conclusions:**

This meta-analysis is the first to comprehensively evaluate the level of circulating spexin in patients with T2DM. Given the substantial heterogeneity observed, the conclusions should be interpreted with caution. Future studies with standardized methodologies are needed to validate our findings and investigate the potential mechanisms.

## Introduction

Diabetes, characterized by chronic hyperglycemia, poses a significant and growing threat to global health. Sustained elevation of blood glucose levels causes structural and functional damage to both the macro- and microvasculature. This underlying pathological process leads to severe complications affecting critical organs and systems, including cardiovascular disease, nephropathy, retinopathy, neuropathy, and diabetic foot ulcers (1). Therefore, elucidating the pathogenesis of diabetes and its complications is of great importance. Early intervention based on such understanding is essential to mitigate the associated decline in quality of life, disability, and mortality.

Studies have shown that dysregulation in the production and/or levels of cytokines can either promote or inhibit the development of diseases such as Type 2 diabetes mellitus (T2DM) and obesity. For instance, low leptin levels are associated with insulin resistance and increased risk of cardiovascular disease (2), whereas higher adiponectin levels can reduce the risk of developing T2DM (3). These findings indicate that different cytokines may exert positive or negative regulatory effects on the development of insulin resistance. Spexin is a multifunctional peptide currently under investigation for its role in metabolic disorders such as obesity and diabetes. Accumulating evidence suggests that spexin participates in cellular energy balance, glucose and lipid metabolism, appetite suppression, and water-electrolyte regulation, and is closely associated with metabolic conditions including obesity, hyperglycemia, and hyperlipidemia (4, 5). Like adiponectin, spexin is reduced in obesity and T2DM, suggesting a protective role. However, unlike leptin—which is adipose-derived and correlates with fat mass—spexin is primarily expressed in neuronal and endocrine tissues and signals via GALR2/3 receptors to modulate both energy intake and expenditure. These distinct properties position spexin as a potentially complementary biomarker beyond established adipokines. However, due to limitations in sample size, ethnic diversity among study populations, and variability in clinical study designs, existing findings on the association between spexin and diabetes remain inconsistent. Some studies have documented a significant reduction in circulating spexin concentrations in individuals diagnosed with T2DM relative to healthy control subjects (6, 7). However, findings from other comparable studies have not consistently aligned with these earlier results (8). Therefore, we conducted a systematic review and meta-analysis to quantitatively assess circulating spexin levels in patients with T2DM compared to healthy controls.

## Methods

### Search

Comprehensive searches were conducted in the following electronic databases: PubMed, Web of Science, OVID, Elsevier Science Direct, and Wiley Online Library. The search strategy was designed to identify studies by combining the term “spexin” with either “Type 2 diabetes mellitus” or “T2DM” in the title or abstract. The following search string was applied: (“spexin”[MeSH Terms] OR “spexin”[All Fields] OR “NPQ”[All Fields] OR “neuropeptide Q”[All Fields] OR “C12ORF39”[All Fields]) AND (“Diabetes Mellitus, Type 2”[MeSH Terms] OR “type 2 diabetes”[All Fields] OR “T2DM”[All Fields] OR “NIDDM”[All Fields] OR “type 2 diabetes mellitus”[All Fields]). All relevant publications from 1980 Jan through 2025 Dec were considered. In addition, reference lists of the retrieved articles were reviewed to identify other potentially eligible studies; however, unpublished reports were excluded. A completed PRISMA checklist is available in the Supplementary Data (**Supplementary Table 1**). The protocol for this systematic review and meta-analysis was registered with PROSPERO (registration number: CRD420251267842).

### Inclusion criteria

Studies were included in the meta-analysis if they met the following criteria: (1) the studies used a case-control or cross-sectional with a distinct control group; (2) the studies reported detailed measurements of spexin levels in patients with T2DM and control groups; (3) the studies were published in English and Chinese.

### Exclusion criteria

Studies were excluded based on the following criteria: unavailability of the full text, duplication of publications, incomplete or non-convertible data, and the implementation of interventions in either the experimental or control groups that deviated from standard diagnostic or management protocols. Furthermore, exclusion applied to studies with significant methodological flaws, as well as non-human research, review articles, conference abstracts, case reports, and editorial commentaries.

### Data extraction and risk of bias

Two investigators independently performed literature screening, data extraction, and cross-validation. Any discrepancies were resolved through discussion or by consulting a third reviewer. Screening began with a review of article titles, followed by the exclusion of irrelevant studies. Subsequently, abstracts and full texts of the remaining articles were examined to assess eligibility for inclusion. When necessary, corresponding authors were contacted via email to obtain missing data. The extracted information included the title, first author, year of publication, study location, sample size, participant age per group, and relevant outcome indicators.

The Newcastle–Ottawa Scale (NOS), which was recommended by the Cochrane Collaboration for assessing the quality of observational studies, was used to evaluate risk of bias (9). Two researchers independently rated each study, compared scores, and resolved inconsistencies through consensus. In cases where agreement could not be reached, a third researcher was consulted to make the final determination. The NOS tool comprises three domains and eight items, with total scores ranging up to 9.

### Statistical analysis

The results of this meta-analysis are expressed as standardized mean differences (SMDs) with 95% confidence intervals (CIs). Between-study heterogeneity was evaluated using Cochran’s Q test and the *I*² statistic. An *I*² value below 50% indicated low-to-moderate heterogeneity, in which case a fixed-effect model was applied; otherwise, a random-effect model was used. Sensitivity analysis was conducted to examine the impact of individual studies on the overall results. Potential publication bias was assessed using Begg’s and Egger’s tests, where a *P*-value of <0.05 was considered statistically significant. All analyses were performed using Stata version 12.0 (StataCorp, College Station, TX, USA).

## Results

**Figure 1:**
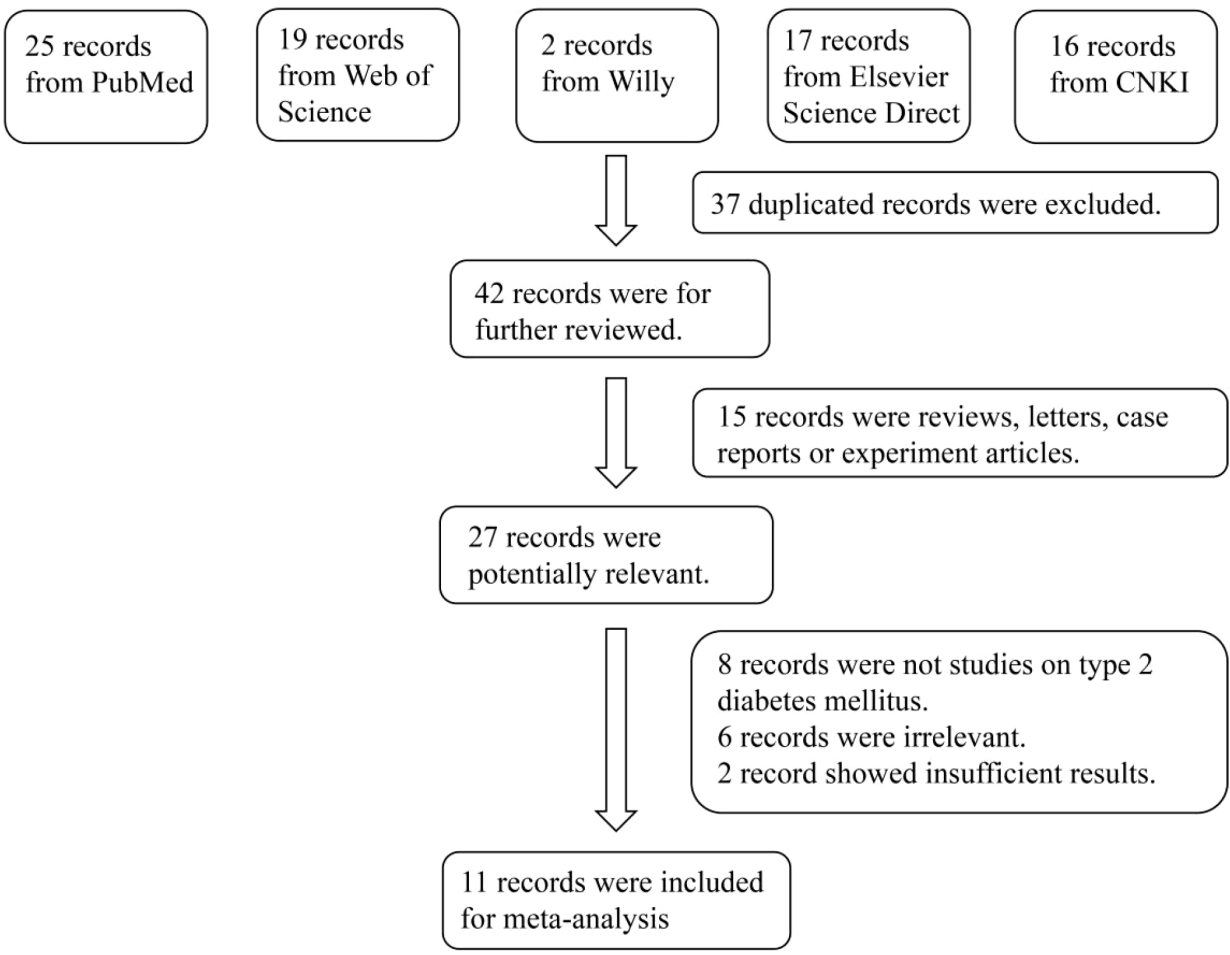
Flowchart of the detailed procedure for the inclusion or exclusion of selected studies.

The initial literature search identified 79 relevant publications from the five electronic databases. Although manual screening of reference lists was conducted, no additional studies met the inclusion criteria. After carefully screening, 11 articles comprising a total of 1,122 cases and 681 controls were included in the final meta-analysis (6–8, 10–17). The flowchart of the study selection process is shown in **Figure 1**, and the key characteristics of the included studies are presented in **Table 1**. All included studies met the criteria for selection, comparability, and exposure categories on the Newcastle–Ottawa Scale for this meta-analysis.

**Table 1:** Study characteristics of the published studies included in the meta-analysis.

Author	Year	Region	Ethnicity	Sample	Number		Spexin		Age		Gender (male/female)	
					Case	Control	Case	Control	Case	Control	Case	Control
Gu L (6)	2015	China	Asian	Serum	121	105	2.04 ± 0.7	3.65 ± 0.73	53.4 ± 8.5	51.1 ± 9.1	60/61	58/47
Hodges SK (10)	2018	USA	Caucasian	Serum	12	10	0.34 ± 0.09	0.38 ± 0.08	16.0 ± 2.1	15.8 ± 2.1	6/6	6/4
Karaca A (7)	2019	Turkey	Caucasian	Serum	30	23	0.65 ± 0.3	0.95 ± 0.33	51 ± 7	33 ± 9	6/24	3/20
Gu L (11)	2020	China	Asian	Serum	88	41	2.18 ± 0.42	3.17 ± 0.56	50.3 ± 10.2	51.76 ± 6.1	22/14	26/15
Khadir A (12)	2020	Kuwait	Caucasian	Plasma	69	66	0.43 ± 0.11	0.44 ± 0.12	52.11 ± 9.43	43.22 ± 12.44	36/33	27/39
Amirpour M (8)	2021	Iran	Caucasian	Serum	168	84	2.67 ± 1.76	2.77 ± 1.70	53.89 ± 8.09	45.06 ± 10.31	51/33	35/49
Tejasw G (13)	2021	India	Caucasian	Serum	220	110	0.57 ± 0.09	0.79 ± 0.03	45.5 ± 10.0	52.8 ± 7.4	——	——
Celik F (14)	2022	Turkey	Caucasian	Serum	60	30	0.44 ± 0.03	0.74 ± 0.05	72.97 ± 5.48	66.81 ± 4.31	33/27	15/15
Dai J (15)	2023	China	Asian	Serum	186	52	0.16 ± 0.06	0.2 ± 0.06	57.04 ± 9.58	49.69 ± 7.86	106/80	17/35
Lin Z (16)	2024	China	Asian	Serum	120	60	2.03 ± 0.21	3.14 ± 0.35	66.95 ± 3.07	66.22 ± 3.01	65/55	33/27
Shamkhi FH (17)	2025	Iraq	Caucasian	Serum	100	100	1.71 ± 0.58	4.4 ± 0.6	56.9 ± 7.9	64.2 ± 2.5	0/100	0/100

### Results of the meta-analysis

**Figure 2:**
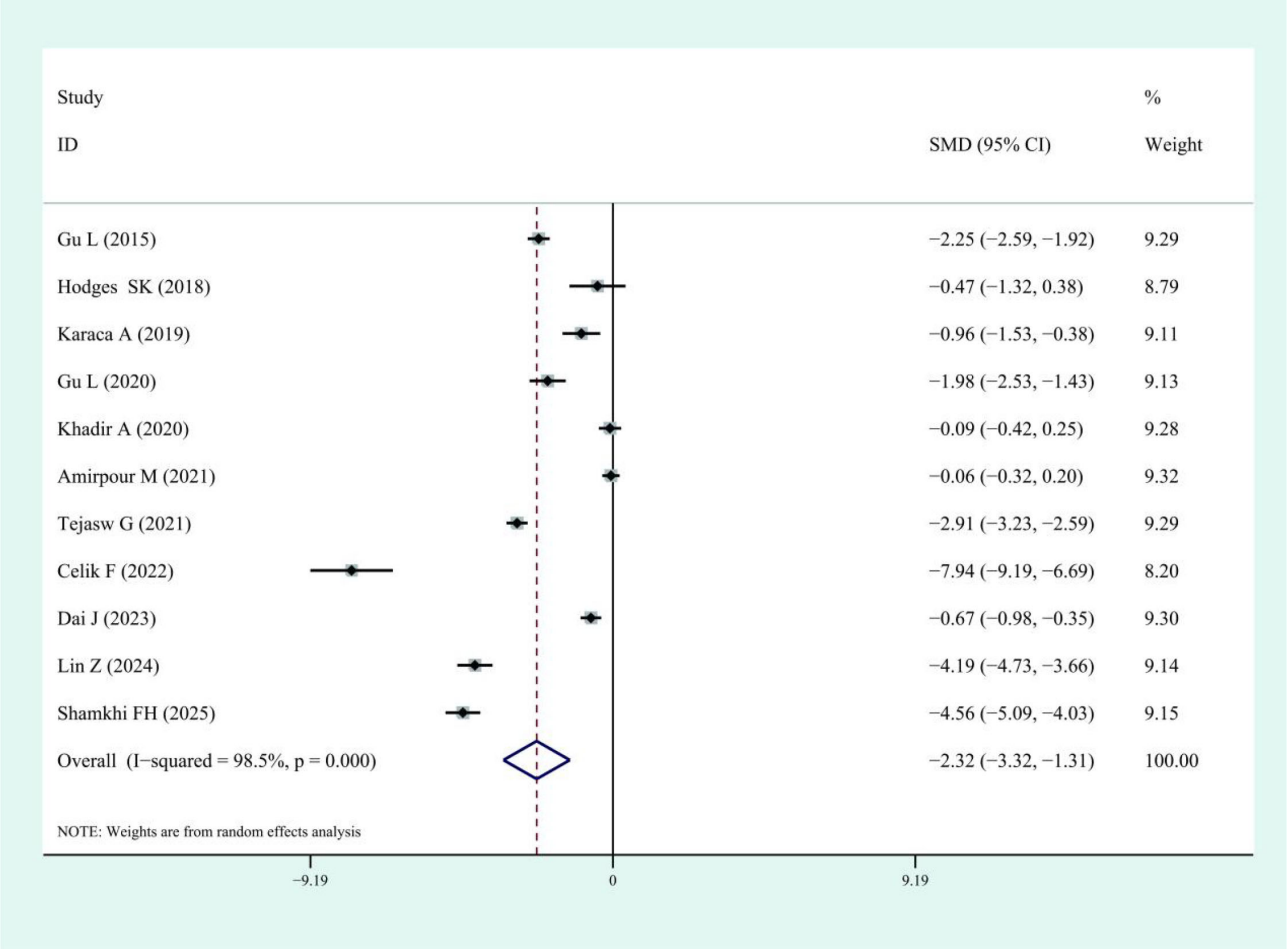
Forest plots of spexin in patients with Type 2 diabetes mellitus compared to the control. Diamond represents the pooled SMDs at 95% CI. SMD, standardized mean difference; CI, confidence interval.

The results of this meta-analysis quantitatively showed a significant decrease in spexin levels among patients with T2DM than the controls (SMD: -2.32, 95% CI: -3.32, -1.31; I^2^ = 98.5%). The forest plots corresponding to these analyses were presented in **Figure 2**. In the present meta-analysis, although the pooled result was statistically significant, considerable heterogeneity was observed among the included studies. To explore potential sources of this heterogeneity, we performed subgroup analyses based on ethnicity, sample source, and sample size. The results across all subgroups were consistent in direction with the main finding; however, none of these subgroup analyses substantially reduced the heterogeneity. Subsequently, we conducted meta-regression with publication year, body mass index, and age as covariates. The meta-regression indicated that none of these covariates were significant moderators of the observed heterogeneity.

### Sensitivity analysis and publication bias

**Figure 3:**
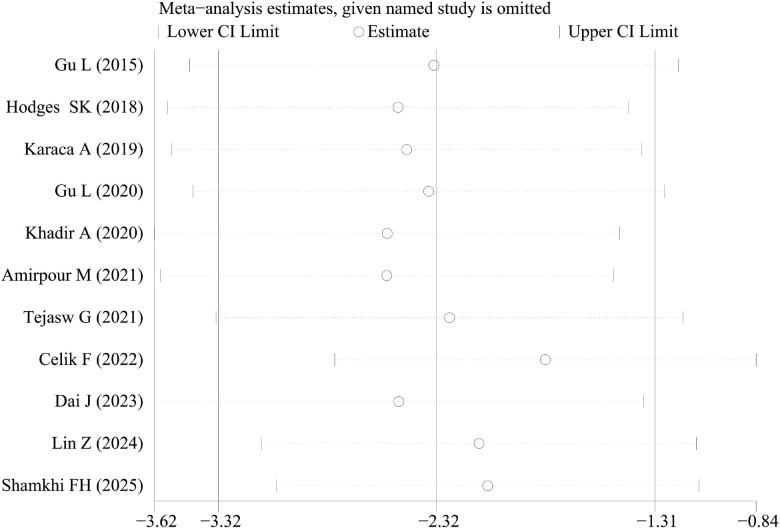
The results of sensitivity analysis.

The removal of any single study in the sensitivity analysis did not substantially alter the pooled effect size, confirming the robustness of our findings (**Figure 3**). To assess potential publication bias, Begg’s and Egger’s statistical tests were performed following our comprehensive literature retrieval. The results from these analyses did not reveal any significant bias in the published literature included in this meta-analysis.

## Discussion

This meta-analysis is the first to systematically evaluate spexin levels in patients with T2DM. Although previous studies have explored the association between spexin and T2DM, their findings have been inconsistent. By synthesizing data from 11 independent studies, this analysis reveals that circulating spexin levels are significantly lower in patients with T2DM than in the control subjects (SMD: -2.32, 95% CI: -3.32, -1.31).

Spexin is a multifunctional peptide that was first discovered by Olivier Mirabeau and colleagues through a Hidden Markov model-based screening of the human proteome. Its gene is located on chromosome 12 and consists of a signal peptide sequence, a mature peptide fragment, and cleavage sites on both sides of the mature peptide (18). Spexin was initially identified in the protein structure of mice and is widely expressed in both the central nervous system and peripheral tissues, including the hypothalamus, cerebral cortex, hippocampus, optic tectum, pons, retina, heart, lungs, liver, thyroid, adrenal glands, muscles, adipose tissue, ovaries, testes, pancreas, and gastrointestinal tract. In the human pancreas, both Spexin mRNA and protein are expressed and are co-localized with insulin within the secretory vesicles of islet cells. This suggests a potential link between the secretion of Spexin and insulin. Studies have found that changes in Spexin levels are regulated by insulin. Variations in either glucose or insulin levels can induce the expression of Spexin mRNA in both the brain and liver, indicating a dual regulatory mechanism (19). Additionally, insulin-related receptors and receptor antagonists can suppress Spexin gene expression. Spexin receptors belong to the galanin (Gal) receptor family, which includes GalR1, GalR2, and GalR3. In vertebrates, Spexin activates endogenous galanin to bind to Gal receptors and couples with Gq/11 to activate protein kinase C, thereby participating in the regulation of insulin sensitivity (20).

Spexin reduces both basal and insulin-stimulated glucose uptake in mouse and human adipocytes (21). Immunofluorescence assays on porcine pancreas confirmed the presence of Spexin within pancreatic tissue and showed that Spexin co-localizes with insulin in pancreatic β-cells. The same study found that Spexin gene expression significantly decreases with increasing glucose concentration, and both gene expression and secretion of Spexin are reduced following insulin treatment (22). These findings suggest that glucose and insulin may regulate the expression and secretion of Spexin. Experiments using goldfish as a model further supported the regulatory role of insulin on Spexin. Feeding was shown to increase plasma levels of glucose, insulin, and Spexin, as well as the expression of insulin and Spexin genes in the liver. Moreover, intraperitoneal injection of glucose and insulin upregulated Spexin mRNA expression in the liver and appetite-controlling brain regions, whereas insulin antagonists and insulin receptor inhibitors significantly suppressed insulin-induced Spexin mRNA expression in the liver and brain, as well as plasma Spexin levels. This suggests a dual regulatory mechanism for insulin on Spexin: on one hand, insulin may activate Spexin gene expression in the liver through autocrine or paracrine signaling; on the other hand, insulin may act as an endocrine signal to stimulate Spexin mRNA expression in the brain (23). However, the potential synergy or antagonism between these pathways remains unclear.

Studies in rats have shown that intraperitoneal injection of Spexin reduces insulin secretion in obese rats. *In vitro* experiments further confirmed that under high glucose conditions, Spexin treatment inhibits insulin secretion in isolated rat islets and INS-1E cells, whereas no such effect was observed under low glucose conditions (24). This indicates that Spexin can modulate insulin secretion both *in vivo* and *in vitro*, and that this regulatory effect is glucose-dependent. Another study in rats also reported that exogenous Spexin treatment decreased insulin levels and reduced the homeostatic model assessment of insulin resistance(HOMA-IR) value in rats fed a high-fat diet, while it had no significant effect on insulin or HOMA-IR in rats fed a normal diet. This study further demonstrated that Spexin dose-dependently inhibited the mRNA expression of Forkhead box transcription factor class O1 (FoxO1), peroxisome proliferator-activated receptor γ coactivator 1α (PGC-1α), phosphoenolpyruvate carboxykinase (PEPCK), and glucose-6-phosphatase (G-6-Pase) (25). These results suggest that Spexin may regulate gluconeogenesis by suppressing the FoxO1/PGC-1α pathway and key enzymes involved in gluconeogenesis (PEPCK and G-6-Pase). Moreover, spexin suppresses food intake through central regulatory mechanisms, primarily involving the hypothalamus. This is achieved by downregulating orexigenic neuropeptides such as neuropeptide Y (NPY), agouti-related peptide (AgRP), and apelin, while simultaneously upregulating anorexigenic factors including proopiomelanocortin (POMC), cocaine- and amphetamine-regulated transcript (CART), melanin-concentrating hormone (MCH), cholecystokinin (CCK), nucleobindin-2 (NUCB-2), and peptide YY (PYY). Evidence supporting this regulatory network has been demonstrated in piscine models (5, 26).

Despite our comprehensive exploration through subgroup analysis and meta-regression, a substantial level of residual heterogeneity persists. This likely reflects the inherent methodological and clinical diversity present in the current body of literature. Firstly, at the methodological level, although all included studies utilized Enzyme-Linked Immunosorbent Assay, variations in commercial kits, specific antibodies, and laboratory protocols may introduce technical variability. Secondly, and perhaps more critically, the clinical population labeled as “diabetes” is inherently heterogeneous, encompassing a wide spectrum of disease duration, therapeutic regimens, glycemic control status, and presence of complications. Unfortunately, most published studies lack granular, stratified data necessary for in-depth secondary analysis on these potential effect modifiers. Consequently, the pooled effect size from our meta-analysis should be interpreted as an estimate of the overarching trend, rather than a precise, universally applicable value for all diabetic patients. This prevailing situation underscores the critical need for future research employing Individual Participant Data (IPD) meta-analysis or large-scale, prospectively designed studies with standardized protocols. Such endeavors are essential to elucidate the true variation patterns of spexin across distinct clinical and demographic subgroups within the diabetic population.

From a clinical translation perspective, although our findings suggest that spexin has potential as an adjunctive biomarker for T2DM risk assessment—particularly for stratifying prediabetic individuals at higher risk of progression—its use as a diagnostic tool is currently precluded by a lack of established cut-off values and standardized detection methods. Furthermore, as an insulin-sensitizing peptide, spexin represents a promising therapeutic target. While research is still in its early stages, spexin-based therapies may eventually offer new options for patients with inadequate responses to conventional treatments. Future clinical studies should investigate whether modulating spexin—either through exogenous supplementation or upregulation of endogenous expression—can improve glycemic control and metabolic outcomes in patients with T2DM.

The extremely high heterogeneity observed in this meta-analysis warrants careful consideration. Several factors may contribute to this substantial variability. First, population characteristics varied considerably across studies, including differences in age distribution, disease duration, glycemic control (HbA1c levels), and medication status. Second, assay-related variability is a major concern: different commercial ELISA kits with varying sensitivities and specificities were used across studies, and sample handling protocols (fasting status, storage conditions) were not standardized. Third, ethnic and geographic diversity may influence baseline spexin levels, as genetic polymorphisms in the spexin gene or its receptors could vary across populations. Fourth, unmeasured confounders such as dietary patterns, physical activity, and comorbid conditions were not consistently controlled for in the original studies. Future research should prioritize methodological standardization and larger, well-phenotyped cohorts to better elucidate the true effect size and reduce heterogeneity.

This meta-analysis, which is the first to quantitatively evaluate spexin levels in patients with T2DM, has several limitations. The overall statistical power was constrained by the predominance of small-scale studies, as large-sample case–control investigations were limited. Furthermore, significant heterogeneity was observed, which could be attributed to the variation in spexin detection methodologies and differences in T2DM severity stages across the included studies. These factors may have influenced the pooled estimates, which indicates that the results should be interpreted with caution and validated in future well-designed research.

## Conclusion

This meta-analysis is the first to comprehensively evaluate the level of circulating spexin in patients with T2DM. The results of this meta-analysis show circulating spexin levels are significantly lower in patients with T2DM than in the control subjects. Given the substantial heterogeneity observed, the conclusions should be interpreted with caution. Future studies with standardized methodologies are needed to validate our findings and investigate the potential mechanisms.

## Data Availability

The original contributions presented in the study are included in the article/**Supplementary Material**. Further inquiries can be directed to the corresponding author.
